# Unraveling Heterogeneity in Transcriptome and Its Regulation Through Single-Cell Multi-Omics Technologies

**DOI:** 10.3389/fgene.2020.00662

**Published:** 2020-07-17

**Authors:** Qiao Rui Xing, Nadia Omega Cipta, Kiyofumi Hamashima, Yih-Cherng Liou, Cheng Gee Koh, Yuin-Han Loh

**Affiliations:** ^1^Epigenetics and Cell Fates Laboratory, Institute of Molecular and Cell Biology, A^∗^STAR, Singapore, Singapore; ^2^School of Biological Sciences, Nanyang Technological University, Singapore, Singapore; ^3^Department of Biological Sciences, National University of Singapore, Singapore, Singapore; ^4^NUS Graduate School for Integrative Sciences and Engineering, National University of Singapore, Singapore, Singapore; ^5^Department of Physiology, Yong Loo Lin School of Medicine, National University of Singapore, Singapore, Singapore

**Keywords:** multimodal single-cell techniques, spatial transcriptome, transcriptome, chromatin accessibility, integrative analysis

## Abstract

Cellular heterogeneity plays a pivotal role in tissue homeostasis and the disease development of multicellular organisms. To deconstruct the heterogeneity, a multitude of single-cell toolkits measuring various cellular contents, including genome, transcriptome, epigenome, and proteome, have been developed. More recently, multi-omics single-cell techniques enable the capture of molecular footprints with a higher resolution by simultaneously profiling various cellular contents within an individual cell. Integrative analysis of multi-omics datasets unravels the relationships between cellular modalities, builds sophisticated regulatory networks, and provides a holistic view of the cell state. In this review, we summarize the major developments in the single-cell field and review the current state-of-the-art single-cell multi-omic techniques and the bioinformatic tools for integrative analysis.

## Introduction

Cellular heterogeneity inherently exists in multicellular organisms, not only among cells of distinct lineages, but also within seemingly identical cells (Altschuler and Wu, 2010; Qi et al., 2014; Buenrostro et al., 2015; Paguirigan et al., 2015). Heterogeneity plays indispensable roles in development, tissue homeostasis, tissue repair and regeneration upon damage, as well as disease progression (Bansal, 2016; Goolam et al., 2016; Chen et al., 2018; Rognoni and Watt, 2018). To elucidate the physiological functions and pathological conditions related to cellular heterogeneity, scientists have dedicated sustained efforts to deconstruct heterogeneity based on certain cellular characteristics. These include anatomical locations, morphological observations, and abundance and localization of bio-molecules involved in the Central Dogma, namely DNA carrying genetic information, intermediate RNA bearing coding information, and the resultant translated protein (Crick, 1970; Wolosewick and Porter, 1977; Gal et al., 2006).

Over the past decade, technical breakthroughs in single-cell toolkits, accompanied with the blossoming of next generation sequencing (NGS), have brought forth an astounding burst in single-cell research (Kumaresan et al., 2008; Tang et al., 2009; Fan et al., 2011; Ramsköld et al., 2012; Wang et al., 2012, 2019; Guo et al., 2013; Sasagawa et al., 2013; Gawad et al., 2014; Smallwood et al., 2014; Buenrostro et al., 2015; Fu et al., 2015; Jin et al., 2015; Klein et al., 2015; Macosko et al., 2015; Rotem et al., 2015; Flyamer et al., 2017; Peterson et al., 2017; Prakadan et al., 2017; Ramani et al., 2017; Stoeckius et al., 2017; Cao et al., 2018; Chen S. et al., 2019; Grosselin et al., 2019; Liu L. et al., 2019; Zhu et al., 2019). In this review, we describe the major achievements in the single-cell field with a focus on multimodal single-cell techniques, particularly spatial transcriptome, and transcriptome and chromatin accessibility. We also review the analytical tools and approaches available for integrative analysis. Lastly, we discuss their potential applications and future directions.

## Major Developments in the Single-Cell Field

The major technical advances in the single-cell field can be summarized as follows: (1) Transition from the targeted measurements to genome-wide profiling. (2) Dramatic increase in the variety of biomolecules assayed. (3) Remarkable increase in the cell throughput. (4) Rise in the number of variables measured within a single-cell. They are discussed in detail in the following paragraphs.

Single-cell genetic techniques involve isolation of individual cells, pre-amplification of genetic materials, and signal detection. Among them, signal detection is the rate-limiting step determining the throughput of data output. Pioneering single-cell techniques relied on primers targeting genes of interest and subsequent measuring of gene expression levels using a polymerase-based qRT-PCR method (Brady et al., 1990; Dulac and Axel, 1995). Later, scientists began to employ hybridization-based microarray chips and digital PCR-based microfluidic chips for signal measurements, which significantly improves the detection throughput (Kamme et al., 2003; Tietjen et al., 2003; Warren et al., 2006). In recent years, advancement in the NGS sequencing platforms allows for profiling of molecular footprints at a genome-wide scale (Kumaresan et al., 2008; Tang et al., 2009; Fan et al., 2011).

Up to date, there is a multitude of single-cell genomic (Kumaresan et al., 2008; Fan et al., 2011; Wang et al., 2012; Gawad et al., 2014; Fu et al., 2015), transcriptomic (Tang et al., 2009; Ramsköld et al., 2012; Sasagawa et al., 2013; Klein et al., 2015; Macosko et al., 2015), and proteomic techniques (Huang et al., 2007; Hughes et al., 2014) developed to de-convolute the heterogeneity based on the copy number variations, genetic mutations, as well as the abundance of transcripts and proteins within each cell. Notably, a wide range of epigenetic mechanisms are involved in the regulation of gene expression, including DNA methylation, histone modification, transcription factor binding, and 3D genomic architecture (Atlasi and Stunnenberg, 2017). However, due to the technical challenges caused by the rarity of genetic materials within individual cells, single-cell epigenetic toolkits were only developed at a later time. To date, there are an array of single-cell methylation techniques developed based on the reduced representation bisulfite sequencing (RRBS) and post-bisulfite adaptor tagging (PBAT) strategies (Guo et al., 2013; Smallwood et al., 2014). A variety of single-cell assays are developed to map the chromatin accessibility landscapes, relying on the differential enzymatic access to the chromatin with varying degrees of openness (Buenrostro et al., 2015; Cusanovich et al., 2015; Jin et al., 2015; Pott, 2017). Droplet-based single-cell ChIP-Seq approaches are developed to map the genetic regions bound by proteins of interest (Rotem et al., 2015; Grosselin et al., 2019; Wang et al., 2019). In spite of the significant improvements to genomic coverage over the years, applications for scChIP-Seq thus far are mostly limited to profiling histone modifications, but not transcription factors which present lesser prevalence in terms of genomic binding. Additionally, a couple of single cell Hi-C methodologies have been developed to examine the heterogeneity in 3D genomic architecture (Flyamer et al., 2017; Ramani et al., 2017).

With the technical explosion, it was soon realized that single-cell libraries suffer from the technical and biological noises, which strongly affects the measurement accuracy. Therefore, the most recent development in the single-cell field is associated with the two strategies proposed to buffer the noises, including an increase in the cell throughput and the number of variables measured within a single cell (Prakadan et al., 2017).

Cell throughput is determined by multiple factors, such as the single-cell platforms utilized, multiplexing strategies applied, and the maximum output of the sequencers. For example, single-cell techniques developed on PCR tubes/plates and valve-based microfluidic chips tend to display lower throughput (magnitude order of 1–3) than that of droplet-based microfluidics and nanowells (magnitude order of 3–6) (Tang et al., 2009; Fan et al., 2011; Macosko et al., 2015; Han et al., 2018). On the other hand, the throughput of plate-based single-cell methods can be boosted up to thousands and millions of cells (magnitude order of 3–7), by applying combinatorial barcode indexing and a few rounds of pool-and-split strategies (Cusanovich et al., 2015; Cao et al., 2017; Zhu et al., 2019). However, in some cases, multiplexing for certain types of libraries, such as genomic DNA libraries, are largely limited by the sequencing capacity of sequencers, as each single-cell library requires high sequencing depth to have full genomic coverage (Prakadan et al., 2017).

The development of multimodal single-cell techniques allows for the measurement of multiple classes of molecules within the same single-cell. Thus far, there are multimodal techniques developed to simultaneously assay transcriptome and genome (Dey et al., 2015; Macaulay et al., 2015), transcriptome and epigenome [transcriptome and DNA methylation (Angermueller et al., 2016; Cheow et al., 2016; Hu et al., 2016; Clark et al., 2018), transcriptome and chromatin accessibility (Cao et al., 2018; Clark et al., 2018; Chen S. et al., 2019; Liu L. et al., 2019; Xing et al., 2019; Zhu et al., 2019)], and transcriptome and protein [limited number of cell surface markers (Peterson et al., 2017; Stoeckius et al., 2017) and intracellular proteins (Genshaft et al., 2016)] within the same single-cell. Addition of temporal and spatial layers on top of transcriptome provides intriguing multi-dimensional insights (Raj et al., 2018; Wang et al., 2018), allowing the connective inspection along the developmental trajectory and anatomical axis. Except for buffering the single-cell sequencing noises, multimodal single-cell techniques enable the correlative investigation between omics and discovery of regulatory relationship across modalities, providing a comprehensive view of cell states.

## Multimodal Single-Cell Techniques

### Spatial Transcriptome

**FIGURE 1:**
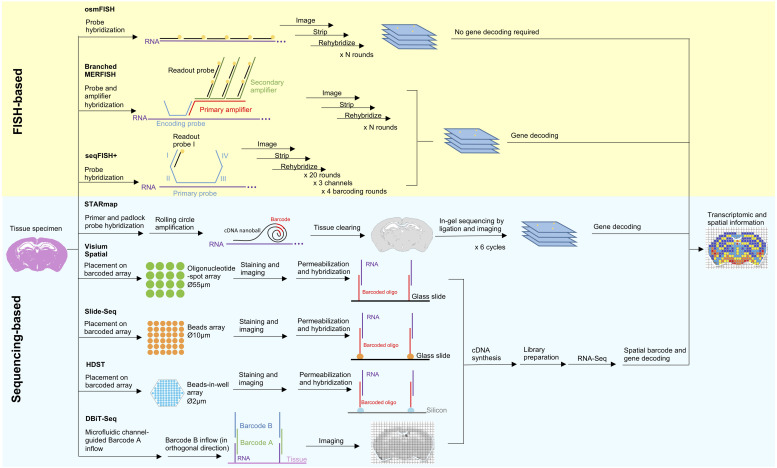
Principles and workflow of recently developed spatial transcriptomic techniques. Two spatial transcriptomic strategies with recent development can be broadly categorized as FISH-based and sequencing-based. FISH-based methods improve on its signal detection (branched MERFISH), diffraction limit (osmFISH and seqFISH+) and gene coverage (seqFISH+). *In situ* sequencing has been combined with tissue clearing technology and modified sequencing by ligation to improve deep tissue visibility and sequencing error in STARmap. Aside from that, many recent techniques are in favor of *in situ* indexing, either by utilizing immobilized (Visium Spatial, HDST, Slide-seq) or flowing (DBiT-seq) barcoded oligonucleotide, followed by *in vitro* sequencing.

Cell physiology relies on the cellular microenvironment and tissue context. However, spatial information is lost under the established single-cell sequencing protocols. Although spatial information can be inferred indirectly by mapping scRNA-Seq data to a fluorescence *in situ* hybridization (FISH)-generated reference map (Achim et al., 2015; Satija et al., 2015), direct approaches are less ambiguous and enable *de novo* discoveries. Various spatially resolved techniques to simultaneously obtain gene expression and spatial information, typically FISH- or sequencing-based, have been reviewed elsewhere (Crosetto et al., 2015; Moor and Itzkovitz, 2017; Strell et al., 2019). Here, we summarize the most recent developments in spatial transcriptomic technologies (Figure 1).

#### Advancement in FISH-Based Spatially Resolved Methods

Quantitation of single mRNA transcript *in situ* can be traced back to single molecule FISH (smFISH) (Femino et al., 1998), however the number of simultaneously identifiable transcripts is limited to a few spectrally distinct fluorophores. Strategies to improve multiplexing include combinatorial labeling (Lubeck and Cai, 2012), sequential hybridization (Lubeck et al., 2014), sequential and serial hybridization (Shah et al., 2016), and multiplexed error-robust (MERFISH) (Chen et al., 2015). Recently, use of branched DNA amplification reportedly improves MERFISH signal detection (Xia et al., 2019). Other challenges in FISH-based approaches include optical crowding due to the large size of fluorescence spots and difficulty in probing short RNA transcripts at multiple distant sites. Cyclic-ouroboros smFISH (osmFISH) is a barcoding- and amplification-free method devised to address these issues at the cost of gene coverage (Codeluppi et al., 2018). More recently, seqFISH+ enables sub-diffraction limit resolution imaging using a 60 “pseudocolor” palette, hence solving the issues of optical crowding, enabling genome-wide targeting, and rendering FISH-based methods capable of *de novo* discoveries for the first time (Eng et al., 2019).

#### Advancement in Sequencing-Based Spatially Resolved Methods

Sequencing-based strategies can be broadly categorized as follows: (1) *in situ* sequencing (ISS), (2) *in situ* indexing, (3) *in vivo* RNA tagging (TIVA) (Lovatt et al., 2014), and (4) serial tissue dissection or single-cell microdissection (Junker et al., 2014; Nichterwitz et al., 2016; Chen et al., 2017). Only the first two strategies are currently undergoing recent development and will be discussed here.

Previously established ISS-based approaches employed rolling circle amplification (RCA) and *in situ* sequencing-by-ligation (SBL) (Ke et al., 2013; Lee et al., 2014). However, these methods suffer from low enzymatic reaction efficiency, limited tissue transparency, and short sequencing reads. Spatially resolved transcript amplicon readout mapping (STARmap) integrates specific RNA amplification, hydrogel-based tissue-clearing, and error-reduced SBL to enable reaction-efficient and 3D RNA sequencing of more than 1000 genes from tissue-slices with a thickness of 150-μm (Wang et al., 2018).

The *in situ* indexing approach pioneered by Ståhl et al. (2016) operates through hybridization of barcoded oligonucleotide-spot array to a permeabilized tissue slice to render spatial coordinates, thereby allowing for the reconstruction of a spatial gene expression map from scRNA-Seq data. However, Ståhl’s method is limited by the spatial resolution of 100 μm, preventing analysis at a single-cell resolution. This technology has been acquired by 10× Genomics and commercialized as Visium Spatial Technology, with improved resolution of 55 μm. On a basis of a similar principle, Slide-seq and high-density spatial transcriptomics (HDST) utilize barcoded bead-array to offer more refined spatial resolutions (10 and 2 μm, respectively), thereby allowing transcriptomic profiling at the single-cell and subcellular levels (Rodriques et al., 2019; Vickovic et al., 2019). A novel microfluidics-based approach known as deterministic barcoding in tissue for spatial omics sequencing (DBiT-seq) indexes tissue *in situ* via the crossflow of two sets of barcodes delivered by parallel microfluidic channels placed orthogonally over the sample in a sequential order (Liu Y et al., 2019). DBiT-seq is highly versatile as it offers a 10 μm spatial resolution and can be extended to detect other biomolecules.

#### Integrative Analysis of Spatial and Transcriptomic Data

Integrated analysis of spatially resolved data and scRNA-Seq data complements each method’s weakness, thus enabling enhanced profiling resolution and accuracy (Moffitt et al., 2018; Zhu et al., 2018; Stuart et al., 2019). Analytical strategy for spatial transcriptomic data typically involves the independent examination of gene expression and subsequent projection back to the spatial map for visualization and inference of cell types and functions. For instance, Trendsceek and SpatialDE are developed to identify genes with spatial expression pattern by incorporating both datasets (Edsgärd et al., 2018; Svensson et al., 2018). The more recent computationally efficient method, Spatial PAttern Recognition via Kernels (SPARK), displays superior statistical power as compared to the previous two methods (Sun et al., 2020).

Aside from spatial expression profiling, spatial transcriptomic studies aim to model cell-cell interactions. Spatial variance component analysis (SVCA) is a computational framework that allows for elucidating the effect of cell–cell interactions to gene expression (Arnol et al., 2019). Another recently available tool known as Multiview Intercellular SpaTial modeling framework (MISTy) is an explainable machine learning framework that models intra- and intercellular views to delineate the relationship between different spatial contexts and gene expression (Tanevski et al., 2020).

The methods described above require a spatial reference map, which is used to formulate a statistical model to infer the probability of the original location of each single cell. Prior to these methods, DistMap (Karaiskos et al., 2017), Seurat v1.1 (Satija et al., 2015), and spatial_mapping (Achim et al., 2015) were developed to formulate the inference models with different computational strategies, which rely on mapping of scRNA-Seq data to the pre-existing FISH-based gene expression atlas. novoSpaRc is a newer method that allows for the *de novo* spatial reconstruction of single-cell transcriptome without prior spatial information (Nitzan et al., 2019). novoSpaRc maps cells to tissues by generalizing this task as an optimal-transport problem under the assumption that physically adjacent cells tend to share similar transcriptome.

#### Beyond Spatial Transcriptomics

Following the advent of spatial transcriptomic era, methods to spatially resolve proteome are also being developed (Angelo et al., 2014; Giesen et al., 2014). Recent improvements include greater multiplexing capabilities (up to thousands of proteins) and reduced input requirements, albeit not at a single-cell level (Xu et al., 2018; Davis et al., 2019). Spatially resolved methods are also moving toward increased versatility. For example, GeoMx^TM^ Digital Spatial Profiler allows for a highly multiplexed and spatially resolved analysis for RNA and DNA or protein. GeoMx^TM^ utilizes antibody or RNA-probe tagged with photocleavable oligonucleotide for *in situ* target binding, accompanied with the region-specific oligonucleotide cleavage and quantification using nCounter^®^, a probe-based direct detection technology (Beechem, 2020). Another method known as APEX-seq/APEX-MS utilizes ascorbate peroxidase APEX2 to probe the spatial organization of the cellular transcriptome and proteome, respectively (Padrón et al., 2019). Furthermore, the road to spatial interactomes has been paved by *in situ* transcriptome accessibility sequencing (INSTA-seq), which allows mapping of RNA and RNA-protein interactions *in situ* (Furth et al., 2019).

To sum up, the earlier FISH-based methods excel over sequencing-based methods in terms of sensitivity and resolution but are limited by targeted detection of only a subset of genes with long transcripts and optical crowding. Improved FISH-based methods theoretically allow for genome-wide profiling at a sub-diffraction limit resolution. Meanwhile, current development of sequencing-based methods is in favor of *in situ* indexing strategies, which are progressing toward subcellular resolution. By harnessing tissue clearing technology and light sheet microscopy, both approaches are advancing from 2D culture to 3D deep tissue profiling. Additionally, simultaneous spatial transcriptomic and proteomic profiling at single-cell level has been accomplished by imaging mass cytometry (Schulz et al., 2018). A thorough 3D spatial multi-omics profiling of complex tissue at genome-wide coverage and subcellular resolution could be achieved in the near future.

### Transcriptome and Chromatin Accessibility

Despite the dedication of sustained efforts to explore epigenetic regulatory mechanisms, limited cues have been decoded due to the complex networks, lack of multimodal toolkits, and insufficient bioinformatics power for integrative analysis. Development of multimodal techniques is tightly associated with the maturity of the stand-alone single-cell techniques. Thus far, among the single-cell epigenetic techniques, assays for DNA methylation are most well-established, followed by those for chromatin accessibility. Single-cell multimodal techniques for transcriptome and DNA methylation were extensively developed until 2018, and comprehensively summarized in a few recent reviews (Chappell et al., 2018; Hu et al., 2018).

#### Single-Cell Approaches for Chromatin Accessibility

**FIGURE 2:**
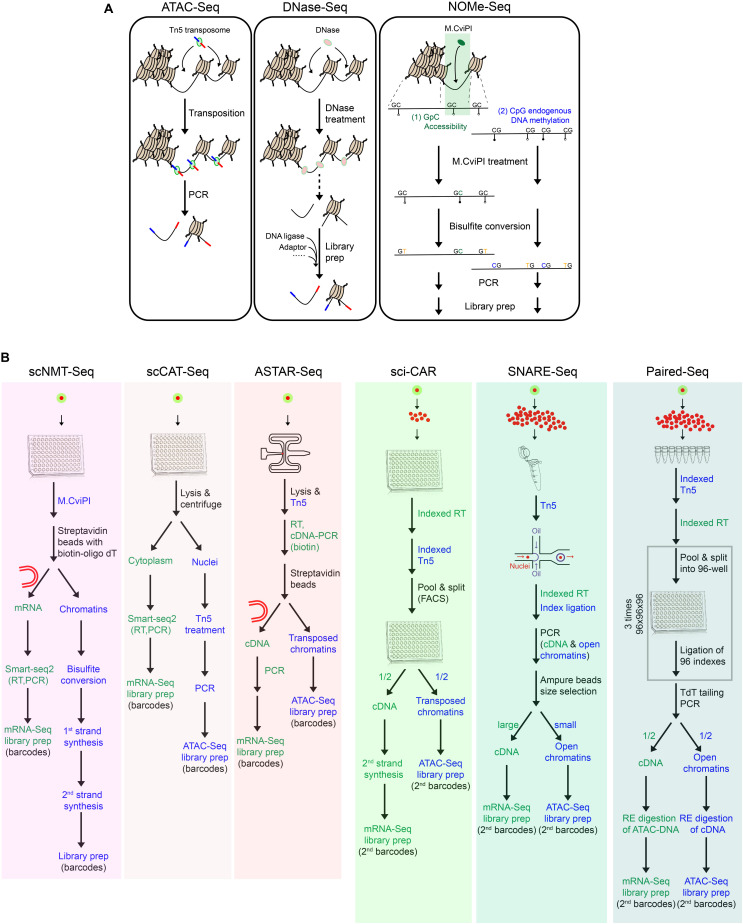
Toolkits for chromatin accessibility and multimodal single-cell techniques. **(A)** Summary of techniques for chromatin accessibility, which have been adapted for single-cell applications. **(B)** Workflow of multimodal single-cell techniques for simultaneous measurement of transcriptome and chromatin accessibility.

Single-cell chromatin accessibility techniques can be categorized into three types, based on the enzymes utilized to enrich open chromatin regions, such as scDNase-Seq with DNase I (Jin et al., 2015), scATAC-Seq with Tn5 transposases (Buenrostro et al., 2015; Cusanovich et al., 2015), and scNOMe-Seq with GpC methyltransferase (M.CviPI) (Pott, 2017; Figure 2A).

scDNase-Seq involves the digestion of accessible chromatin regions with DNase I, followed by ligation of sequencing adaptors (Jin et al., 2015). scATAC-Seq employs a one-step cut-and-paste mechanism, where Tn5 cuts the open chromatin and inserts its adaptor payload sequences simultaneously (Buenrostro et al., 2015; Cusanovich et al., 2015). In comparison, due to its protocol simplicity, scATAC-Seq is more suited for high-throughput applications. Different from these fragmentation-based methods, scNOMe-Seq utilizes GpC methyltransferase (M.CviPI) to methylate cytosine in the accessible GpC dinucleotides, followed by bisulfite sequencing (Pott, 2017). Downstream analysis can differentiate the methylated GpCs from the methylated CpGs, which represent signals of the accessible/nucleosome-depleted regions and the endogenously methylated DNA, respectively.

There are pros and cons of scNOMe-Seq over the fragmentation-based methods. Advantages of scNOMe-seq are as follows: (1) It provides bi-layer information of chromatin accessibility and DNA methylation within the same cell. (2) It presents the comprehensive accessibility status across the entire genomic regions, including both open and closed regions. Comparatively, fragmentation-based methods only map the open chromatin, while the undetected regions could be either close chromatin or open chromatin which was lost during the process. (3) It captures high resolution accessibility landscapes. Notably, GpC sites prevalently exist within the genome (∼1 in 16 bp), resulting in its high genomic resolution of around 16 bp, as compared to that of scATAC-Seq and scDNase-Seq (100–200 bp). Disadvantages of scNOMe-Seq are as follows: (1) The library suffers from low quality and high background noises, owing to the bisulfite treatment. (2) It excludes GpCpG sequences from the downstream analysis, which comprises 20% of the genome. This is associated with the inherent principle of the method to differentiate chromatin accessibility (GpC) from the endogenous methylation status (CpG). (3) It requires high sequencing depth to have full genome coverage, restricting its application for high-throughput profiling.

#### Multi-Modal Single-Cell Techniques for Transcriptome and Chromatin Accessibility

Up to date, there are six multimodal techniques jointly profiling chromatin accessibility and transcriptome within a single-cell or single-nucleus (Figure 2B). Different from the others, scNMT-Seq adopts methyltransferase-based chromatin accessibility approaches and simultaneously profiles the whole-cell transcriptome (Clark et al., 2018; Figure 2B). Briefly, single-cells are first treated with GpC methyltransferase (M.CviPI), after which mRNA is enriched with oligo-dT beads and subjected to library preparation following Smart-seq2. Meanwhile, the isolated DNA is treated with bisulfite, followed by adaptor ligation and library preparation.

The others employ Tn5 transposases for chromatin accessibility, among which scCAT-Seq and ASTAR-Seq demonstrate high detection sensitivity (Figure 2B). scCAT-Seq (Liu L. et al., 2019) is a plate-based method, which involves the physical separation of cytoplasmic RNA from nuclear DNA by combining mild lysis and centrifugation (Figure 2B). mRNA is then collected from the supernatant and subjected to library preparation following Smart-seq2 protocol. Meanwhile, precipitant nucleus DNA is subjected to Tn5 transposition and subsequent library preparation. In comparison, ASTAR-Seq employs the automated programmable valve-based microfluidic chips (Xing et al., 2019), on which open chromatins are tagmented with Tn5, followed by reverse transcription of mRNA within the whole-cell (Figure 2B). In the following steps, cDNA is labeled with biotin during PCR amplification and separated from the open chromatins using streptavidin beads.

High-throughput bimodal methods jointly profile transcriptome and chromatin accessibility within single-nucleus through two different strategies and platforms, including droplet-based microfluidics (SNARE-Seq) and plate-based combinatorial indexing (sci-CAR and Paired-Seq) (Figure 2B).

In SNARE-Seq protocol, nuclei are first extracted, permeabilized, and tagmented with Tn5 in bulk (Chen S. et al., 2019; Figure 2B). The single tagmented nucleus is then encapsulated in droplets along with the barcoded beads and splint oligo which is designed to label the cDNA and accessible regions in a single-cell with the same barcode. Nuclei are then pooled, and the cDNA and accessible chromatins containing cell-specific barcodes are segregated by beads purification based on their size difference.

On the contrary, sci-CAR applies the reverse reaction order of *in situ* reverse transcription followed by chromatin tagmentation and adopts a combinatorial indexing approach to incorporate cell-specific barcodes (Cao et al., 2018; Figure 2B). Briefly, 5000 permeabilized nuclei are first sorted into each well of a 96-well plate, in which single-strand cDNAs and transposed open chromatins are generated and labeled with different sets of oligos carrying well-specific barcodes. All nuclei are pooled, 25 of which are then re-distributed into each well of a 96-well plate by FACS for second strand synthesis of cDNA. Afterward, materials within each well are dedicated to RNA and DNA portions for independent library preparations, during which the second indexes are introduced.

Paired-seq, an ultra-high-throughput bimodal technique developed on the basis of sci-CAR, adopts ligation-based combinatorial indexing strategy to simultaneously tag cDNA and open chromatin fragments with the same barcodes (Zhu et al., 2019; Figure 2B). Paired-seq can achieve a throughput of one to ten million. Contrary to the reaction order of sci-CAR, tagmentation is first performed on 250,000 permeabilized nuclei in eight replicates, followed by reverse transcription of mRNA. All nuclei are then subjected to three rounds of pool-split-ligation, where nuclei are equally distributed into each well of a 96-well plate and well-specific barcodes are ligated to both cDNA and accessible chromatins. After TdT-mediated second strand synthesis of cDNA, both classes of molecules are amplified by PCR and then dedicated to two portions. To remove contaminant biomolecules from the other class, each aliquot is treated with different restriction enzymes specifically digesting the pre-designed sites in Tn5 adaptors and RT primers, prior to mRNA-Seq and ATAC-Seq library preparation, respectively.

In sum, given the lower cell throughput, scNMT-Seq, scCAT-Seq, and ASTAR-Seq libraries display higher sensitivity in detecting full-length transcripts and accessible regions. Specifically, scNMT-Seq presents the highest coverage for accessible chromatins at the cost of sequencing. scCAT-Seq and ASTAR-Seq are comparable in terms of the complexity and sensitivity of accessible regions, whereas ASTAR-Seq demonstrates superior gene detection sensitivity than scCAT-Seq, which might be due to its mRNA capture from whole-cell, instead of only cytoplasmic compartments. On the other hand, high-throughput multimodal methods assay the nuclear transcriptome with the bias toward the 3′ end of transcripts. Their scRNA-Seq libraries are of similar quality (UMI: 1100–1800; genes: 400–800), whereas SNARE-Seq and Paired-Seq exhibit higher chromatin complexity than sci-CAR (Sites No.:1500–2600 vs. 200–500). This might be associated with its reaction order of reverse transcription followed by tagmentation, resulting in loss of chromatin architecture during the prolonged incubation of chromatin. Altogether, the appropriate multimodal techniques should be carefully chosen, weighing the delicate balance of various factors, including throughput, sensitivity, and the qualities of single-cell libraries generated.

#### Integrative Analysis of Transcriptome and Chromatin Accessibility

As compared to uni-modal single-cell datasets, multimodal single-cell libraries for chromatin accessibility and transcriptome present remarkable advantages, which are categorized into the following groups. Additionally, we discuss the analytical approaches and toolkits employed for integration of chromatin accessibility and transcriptome.

(1) Refined classification enabling the identification of rare cell types. There are a few strategies to achieve integrative clustering. Instead of clustering based on the highly accessible regions, chromatin accessibility signals of the same cell-types or clusters inferred from scRNA-Seq are first merged, followed by peaks calling, projection onto a low dimensional space, and identification of clusters (Cao et al., 2018; Chen S. et al., 2019). Besides, computational algorithms, such as coupled non-negative matrix factorizations (NMF), are devised specifically to cluster cells, based on the simultaneous evaluation of gene expression and chromatin accessibility and the correlation between modalities (Huebschmann et al., 2017; Liu L. et al., 2019; Xing et al., 2019). NMF identifies gene-peak pairs with differential expression and accessibility across the NMF clusters. In addition, SnapATAC, a software designed for stand-alone single-nucleus ATAC-Seq libraries, is adapted for analyzing multimodal scRNA-Seq and scATAC-Seq libraries (Fang et al., 2019; Zhu et al., 2019). In the adapted SnapATAC pipeline, a cells-to-bins DNA matrix and a cells-to-genes RNA matrix are generated separately and computed into an integrative matrix, which are then subjected to dimensionality reduction and graph-based clustering. Generally, clustering based on the multimodal single-cell libraries generates more distinct boundaries between cell types and identifies rare populations which are largely undetected in the stand-alone scATAC-Seq libraries. For example, clustering of SNARE-Seq libraries based on chromatin accessibility with prior knowledge of cell type identities from its linked transcriptomic profiles, sensitively detects rare cell types within mouse neonatal cerebral cortex and the accessible chromatin regions specific to them (Chen S. et al., 2019).
(2) Modality correlation along the constructed trajectories. Despite similarities in transcriptomic profiles, cells may be primed for development in different degrees at the epigenetic level (Lara-Astiaso et al., 2014). To identify minority cells with differential developmental status between modalities, cellular positions can be compared in the trajectories, which are independently constructed based on single-cell transcriptome and chromatin accessibility signals. In addition, the temporal order of dynamics in accessibility and expression can be perceived for genes of interest (Cao et al., 2018; Clark et al., 2018; Chen S. et al., 2019). Moreover, TFs critical for development can be identified by overlapping TF motifs with differential chromatin accessibility and TFs with differential expression across the pseudotemporal axis (Zhu et al., 2019).
(3) Annotation of putative target genes for the cis-regulatory elements (CRE). A multitude of CREs which demonstrate tissue-specific activities have been identified in the mammalian genome (Heintzman et al., 2009; Ong and Corces, 2011). However, annotation of their putative targets remains a challenge, due to the complicated regulatory networks. For example, CRE-to-gene regulation can be one-to-one, one-to-many, and many-to-one, and CREs for each target vary across the cell types. In addition, putative targets of CREs are not absolutely determined by the genomic distance between them. Using multimodal single-cell libraries, interactions of CRE-to-gene can be inferred based on their co-accessibility, which can be further overlapped with the gene expression. Noteworthy, bimodal single-cell datasets improve the prediction accuracy of putative targets by 4–5 times (Cao et al., 2018).

### Computational Integration of Multi-Modal Single-Cell Datasets

In line with their superior maturity in the technique development, algorithms and analytical tools for unimodal single-cell libraries are much more diverse, which have been reviewed extensively elsewhere (Chen G. et al., 2019; Luecken and Theis, 2019; Wu and Zhang, 2020). Here we review the bioinformatic toolkits developed for the integration of single-cell libraries across various modalities.

With the rapid growth of single-cell libraries prepared for Cell Atlas programs, integration of scRNA-Seq and genomic-based single-cell libraries is of utmost importance. To annotate the cell types in the genomic-based single-cell datasets, various analytical approaches and softwares have been developed. For instance, a reference-guided approach allows for pairing of scRNA-Seq and scATAC-Seq, by fitting a linear model to match the global variations in chromatin accessibility and transcriptome depicted by bulk ATAC-Seq and RNA-Seq libraries, respectively (Buenrostro et al., 2018). A k-nearest neighbor (KNN)-based classification approach is employed to transfer cell-type labels of the nearest scRNA-Seq neighbors to sci-ATAC-Seq libraries (Cusanovich et al., 2018). Likewise, Seurat v3 allows for the classification of scATAC-Seq data based on the cell types determined from scRNA-Seq data of a similar sample (Stuart et al., 2019).

Apart from cell annotation, the most commonly adopted integration strategy is to identify correspondence between features of distinct modalities. For example, clonealign integrates scRNA-Seq and scDNA-Seq data on the basis of an assumed positive correlation between copy number and gene expression, which is used to analyze the clone-specific biological pathways in human cancers (Campbell et al., 2019). Gradient-boosting model (GBM) integrates the scDrop-Seq and scTHS-Seq libraries of brain tissue, by predicting differentially accessible sites based on the differentially expressed genes and vice versa (Lake et al., 2018). In addition, NMF (Huebschmann et al., 2017) and Coupled NMF (Duren et al., 2018) can be adopted for the integration of chromatin accessibility and transcriptome. Similarly, the integrative non-negative matrix factorization (iNMF) method, also known as LIGER, integrates DNA methylation and transcriptome, based on the negative correlation between gene expression and gene-body methylation (Welch et al., 2019). Likewise, the multi-omics factor analysis (MOFA) method explicates the associations between DNA methylation states and transcriptional profiles within the same cell (Argelaguet et al., 2018).

Without prior knowledge of feature correspondence, integration between modalities can be achieved by finding the common biological states, which is based on the assumption that cells of the same type or state share correlations across modalities. For example, MATCHER projects cells from different experiments onto a common 1D pseudotime space under the assumption that a common developmental trajectory similarly affects both modalities (Welch et al., 2017). MATCHER was applied to study the temporal dynamics of transcriptome and DNA methylation during iPSCs reprogramming (Angermueller et al., 2016).

## Perspectives and Future Directions

As the desire to gain a holistic view of cells grows, single-cell fields are advancing toward the invention of multi-omics techniques and integrative data analysis across modalities. The achievements made over the past decades are laudable, but many challenges still remain to be overcome: (1) Sensitivity of the multimodal techniques is often lower than the corresponding stand-alone single-cell techniques. This is likely due to sample loss during isolation of various classes of molecules, or degradation of molecules due to the incompatibility of protocols. Extensive optimizations should be carried out for multi-modal single-cell protocols to increase coverage, reduce dropout rate, and maximize signal-to-noise ratio. (2) More multi-omic techniques have yet to be developed. Due to the technical challenges, limited single-cell techniques are available for some applications, such as proteomics, chromatin structure, and chromatin immunoprecipitation. Multi-omic techniques combining them with transcriptome would undoubtedly provide novel insights into transcriptional and translational regulatory mechanisms. (3) On top of that, the number of omics should be further increased to achieve better correlation across modalities. The ultimate goal is to develop multi-omic techniques enabling collection of information from all omics at a single-cell resolution. (4) Bioinformatic tools tailored for multi-modal libraries would facilitate the integration across modalities and leverage the comprehensive characterizations of cell states. Altogether, single-cell omics provide unprecedented opportunities to investigate crucial biological questions in multi-dimensional spaces.

## Author Contributions

QX, NC, and Y-HL conceived the structure of review. QX, NC, and KH wrote the review. Y-CL, CK, and Y-HL read and edited the review. All authors contributed to the article and approved the submitted version.

## Conflict of Interest

The authors declare that the research was conducted in the absence of any commercial or financial relationships that could be construed as a potential conflict of interest.
